# Expanding High‐Fidelity Multiplexing in Ultrasensitive Single‐Molecule Protein Detection via Proximity Barcoding

**DOI:** 10.1002/anie.9011937

**Published:** 2026-05-28

**Authors:** Chi‐Chia Wang, Emily Dorsey, Connie Wu

**Affiliations:** ^1^ Life Sciences Institute University of Michigan Ann Arbor Michigan USA; ^2^ Department of Biomedical Engineering University of Michigan Ann Arbor Michigan USA; ^3^ Department of Pharmaceutical Sciences University of Michigan Ann Arbor Michigan USA

**Keywords:** biomarkers, digital ELISA, multiplexing, single molecule, ultrasensitive protein detection

## Abstract

The human proteome presents a vast information reservoir for basic and diagnostics research, yet the low abundances of many proteins in biofluids pose an analytical challenge. While ultrasensitive methods such as digital enzyme‐linked immunosorbent assay have expanded the window of detectable proteins, multiplexing with high accuracy, sensitivity, and throughput remains limited by cross‐reactivity and signal readout channels. To address this challenge, we introduce PRO‐MOSAIX (PROximity‐barcoded Molecular On‐bead Signal Amplification for Individual MultipleXing), a high‐accuracy multiplex digital immunoassay platform that integrates ultrasensitive single‐molecule protein detection with proximity ligation. PRO‐MOSAIX generates “ON” signals only from matched affinity reagents in proximity, minimizing false positives from cross‐reactive binding. This approach overcomes the multiplexing ceiling imposed by fluorescence spectral overlap by employing a single signal readout channel and DNA barcoding. We further improve multiplexing fidelity by mitigating a secondary source of false positives from DNA‐based signal amplification. As a proof of principle, we establish and validate a 15‐plex PRO‐MOSAIX assay in human plasma, with low femtomolar sensitivities and high measurement accuracies. PRO‐MOSAIX is modular and utilizes common laboratory instrumentation with a high‐throughput flow cytometric readout, providing a broadly accessible tool across research and clinical labs and bridging the gap between analytical sensitivity and high‐order multiplexing.

## Graphical abstract

We develop an ultrasensitive, high‐accuracy multiplex protein detection platform integrating high‐throughput single‐molecule protein detection with proximity ligation. Using paired DNA barcodes, this platform breaks the digital immunoassay multiplexing ceiling imposed by cross‐reactivity and fluorescence spectral overlap, providing a broadly accessible tool that bridges the gap between analytical sensitivity and multiplexing.

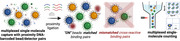

## 1 Introduction

The extraordinary complexity of the human proteome presents a wealth of biological information for understanding and diagnosing diseases. However, much of this potential remains untapped due to measurement technology limitations and fundamental gaps in protein biomarker identification. Human plasma alone contains thousands of proteins spanning over 10 orders of magnitude in concentration [1, 2], but only a small fraction is routinely used in the clinic, in part due to challenges in identifying biomarkers with high sensitivities and specificities [3, 4, 5]. Many low‐abundance proteins in plasma and other biofluids remain inaccessible by traditional detection methods such as enzyme‐linked immunosorbent assay (ELISA), which have low to sub‐picomolar (pM; 10^−12^ M) detection limits. Another major challenge is the simultaneous detection of many proteins with both high sensitivity and accuracy. Such multiplexing capabilities are critical for capturing disease complexities and heterogeneities, improving measurement throughput, cost‐effectiveness, and sample conservation.

Ultrasensitive detection methods such as digital ELISA have expanded the window of detectable proteins by three to four orders of magnitude over conventional ELISA in recent years, reaching low‐abundance biomarkers such as many cytokines, tumor‐derived or tumor‐enriched proteins, and neurodegenerative disease biomarkers [6, 7, 8, 9, 10]. In digital ELISA, single molecules are captured on a high excess number of antibody‐coated paramagnetic beads, ensuring a Poisson distribution in which most beads carry one or zero molecules. Single beads are then isolated into individual femtoliter microwells or droplets for signal amplification from single molecules, enabling counting of “ON” and “OFF” beads. We recently developed a compartmentalization‐free digital ELISA platform, Molecular On‐bead Signal Amplification for Individual Counting (MOSAIC), which generates a fluorescent signal localized to each bead carrying a single target molecule via rolling circle amplification (RCA) [9]. This localized signal amplification enables high‐throughput readout via flow cytometry and achieves low‐ to mid‐attomolar (aM; 10^−18^ M) detection limits, enhancing sensitivity by an order of magnitude over conventional digital ELISA.

**FIGURE 1:**
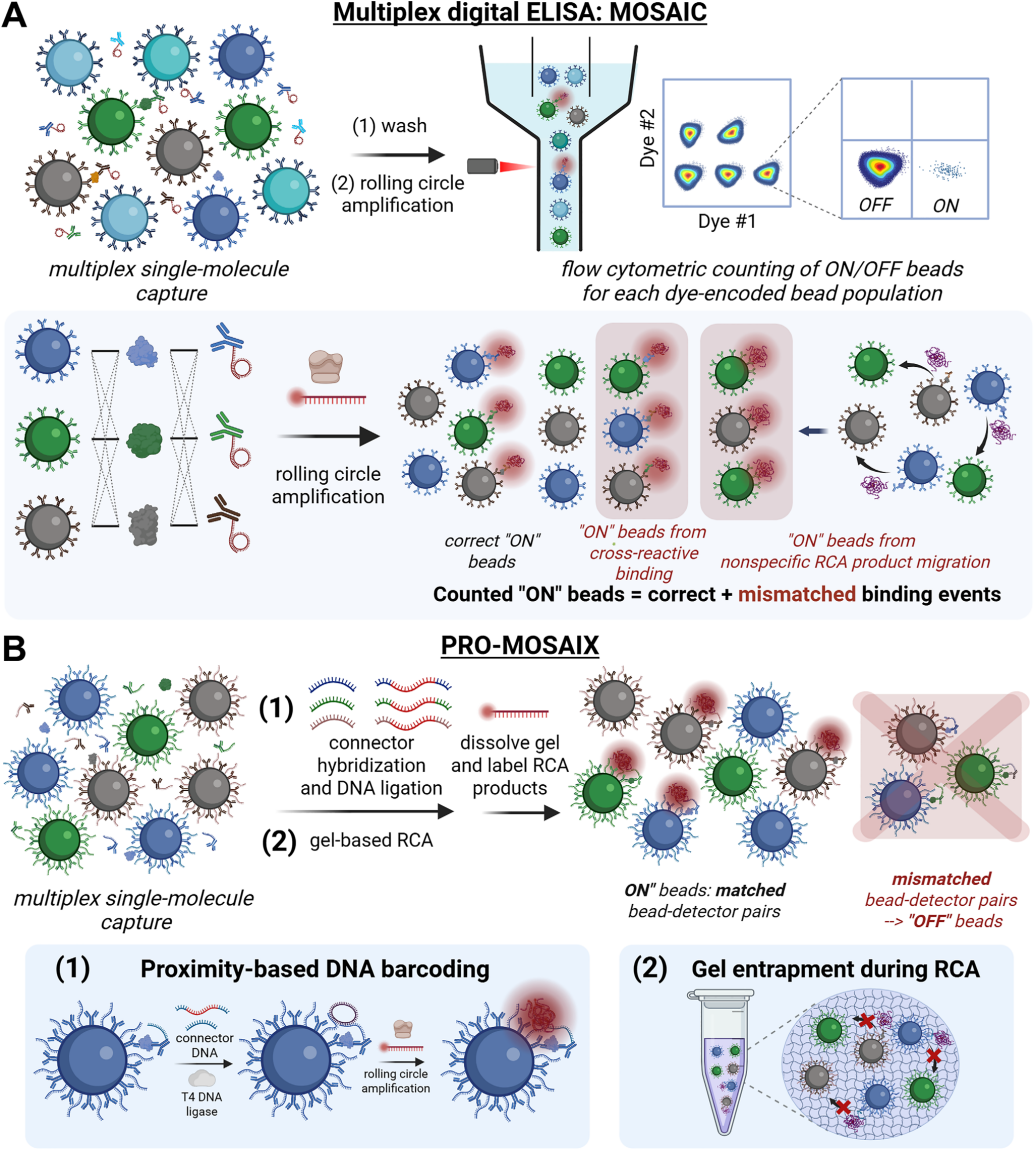
Multiplex digital immunoassay workflows and sources of cross‐reactive signals. (A) Multiplex single‐molecule protein detection with MOSAIC. Single immunocomplex sandwiches are formed on a mixture of unique fluorescent dye‐encoded beads, each coated with capture antibodies for a specific analyte. Upon signal amplification via RCA, fluorescent “ON” and “OFF” beads are counted for each bead population by flow cytometry. Cross‐reactive interactions between different antibodies and analytes lead to mismatched binding events that undergo signal amplification, yielding incorrect “ON” beads. False positive “ON” beads can also result from nonspecific DNA product diffusion across beads during RCA. (B) Schematic of PRO‐MOSAIX workflow. Each unique fluorescent dye‐encoded bead is co‐coupled with capture antibody and a proximity DNA barcode, while the detector antibody for each analyte is conjugated to a paired proximity DNA barcode. Upon multiplex single‐molecule capture, only correctly formed single immunocomplex sandwiches will template the circularization of a DNA template after addition of a connector oligo mixture and DNA ligase (1: proximity‐based barcoding). Each connector oligo set contains a shared sequence for hybridization of the same fluorescently labeled DNA probe after RCA. Nonspecific DNA product diffusion during signal amplification is prevented by encapsulating beads in an agarose gel during RCA (2: gel entrapment during RCA), after which the gel is dissolved for bead retrieval, labeling with fluorescent DNA probe, and flow cytometric counting. Figure was created with BioRender.

Despite advances in sensitivity, accurate multiplexing in digital ELISA remains severely restricted by cross‐reactivity and limited signal output channels, with up to only eight‐plex digital ELISA assays reported to date [9, 11, 12]. Multiplexing is achieved using unique fluorescent dye‐encoded capture beads for each analyte, but exponentially increasing combinatorial interactions between affinity reagents and analytes with increased multiplexing inevitably lead to cross‐reactive binding and false‐positive “ON” signals (Figure 1A). Several approaches have been developed to mitigate cross‐reactivity in digital ELISA, including a temporal separation method in which sequential sample incubation with each bead type and then corresponding detector antibody eliminates cross‐reactive “ON” signals [13]. However, the increased assay time and workflow complexity with each additional analyte impose a practical limit on multiplexing order. More recently, we developed a barcoded MOSAIC platform, where each detector antibody is conjugated to a DNA template corresponding to a unique fluorescent DNA probe for RCA product labeling [11]. Correct capture‐detector binding combinations are identified via matched bead and “ON” probe fluorescent colors, while mismatched color pairs from cross‐reactive binding events are not counted. Although this strategy increases accuracy in multiplex digital ELISA, multiplexing capacity remains limited by the number of unique probe colors due to fluorescence spectral overlap, with up to only five unique barcoding combinations reported.

Cross‐reactivity also poses a major barrier in other multiplex immunoassays [14]. Various strategies have been employed to mitigate cross‐reactivity in multiplex immunoassays, including spatial or temporal separation of affinity reagents [15, 16, 17, 18] and proximity‐based approaches requiring matched affinity reagent binding for signal generation [19, 20, 21]. However, reliable and scalable detection of low‐abundance proteins in biofluids requires the integration of ultrasensitive detection, robust multiplexing capabilities, and streamlined workflows. While advances in proteomics and multiplex protein detection platforms have enabled simultaneous measurements of tens to thousands of proteins, current methods remain limited by tradeoffs among sensitivity, multiplex accuracy, workflow complexity, and specialized instrumentation requirements [5, 22, 23]. Consequently, a critical gap persists between the exceptional sensitivity of digital ELISA, high multiplex capacity and accuracy, and broad accessibility across standard laboratory settings.

To bridge this technological gap and break the multiplexing ceiling in ultrasensitive single‐molecule protein detection, we introduce PRO‐MOSAIX, a modular platform that enables high‐order multiplexing in digital ELISA with minimal cross‐reactivities. PRO‐MOSAIX integrates the exceptional sensitivity, high‐throughput readout, and broad laboratory accessibility of MOSAIC with the versatility of DNA barcoding and proximity ligation, where each capture bead and the corresponding detector antibody are barcoded with a unique proximity oligo pair (Figure 1B). When in proximity in a correct immunocomplex sandwich, the matched DNA barcodes template the circularization and ligation of connector DNA oligos for RCA. Thus, only matched capture and detector reagents bound in a correct immunocomplex sandwich will generate an “ON” signal. Importantly, this approach uses a single signal output channel by encoding a common sequence for fluorescent DNA probe hybridization across all connector oligos, bypassing the multiplexing limit imposed by fluorescence spectral overlap. In conjunction, we identify an additional source of false positive signals in multiplex MOSAIC assays, arising from nonspecific DNA product diffusion between beads during signal amplification (Figure 1A). This phenomenon is addressed via reversible agarose gel encapsulation of beads during RCA to physically trap DNA products. Integrating the proximity barcoding and gel entrapment approaches vastly reduces cross‐reactive signals in multiplex single‐molecule protein detection. As a proof‐of‐principle, we developed and validated an ultrasensitive 15‐plex PRO‐MOSAIX assay with high measurement accuracies across low‐ and medium‐abundance cytokine and tumor biomarkers in plasma. Our results establish PRO‐MOSAIX as a high‐fidelity multiplex digital immunoassay platform using common laboratory instrumentation, laying the foundation for expanded multiplexing in ultrasensitive protein profiling across diagnostic and fundamental applications.

## 2 Results and Discussion

### 2.1 Development of Gel Entrapment Strategy to Mitigate off‐Target on Signals during DNA Amplification

**FIGURE 2:**
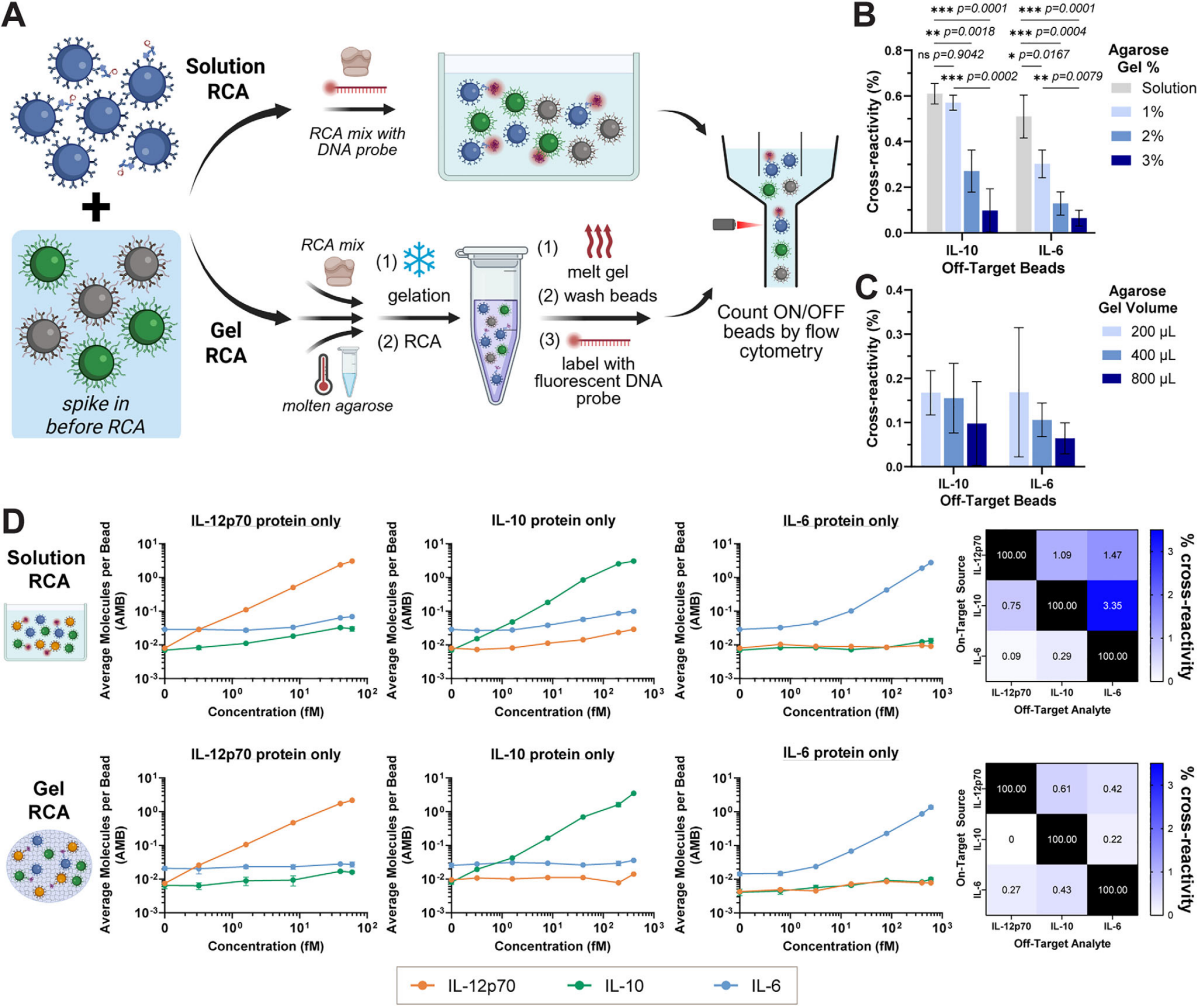
Identification of false positive signals arising from the DNA amplification process in MOSAIC‐based assays. (A) Schematic of spike‐in controls in which off‐target antibody/DNA co‐coupled dye‐encoded beads were added to beads carrying circular DNA template‐labeled immunocomplexes, immediately before solution‐ or gel‐based RCA. For solution‐based RCA, beads were incubated with an RCA mixture containing phi29 polymerase and fluorescent DNA probe, and amplification proceeded in plates. For gel‐based RCA, beads were transferred to tubes, resuspended in RCA mix without fluorescent probe, mixed with an equal volume of molten agarose, and solidified on ice. Amplification occurred within the gel, after which the beads were recovered by melting the gel at high temperature and labeled with the probe. (B,C) Effects of agarose gel density (B) and volume (C) on false positive ON bead signals caused by nonspecific migration of DNA products during RCA. IL‐12p70 beads (30,000 beads) and detector antibody were incubated with 30 fM IL‐12p70, with 30,000 each of off‐target IL‐10 and IL‐6 beads spiked in immediately prior to RCA for all conditions. Cross‐reactivity (%) is defined as the ratio of off‐target/on‐target bead AMB values after background subtraction. The data represent the mean ± standard deviation of *n* = 3 technical replicates and were analyzed by one‐way ANOVA with Tukey's post‐hoc test for multiple comparisons. *p<0.05; **p<0.01; ***p<0.001. There were no statistically significant differences among gel volumes (C). (D) Protein dropout curves across increasing concentrations of individual target proteins to evaluate cross‐reactivities in a three‐plex MOSAIC assay with solution‐ and gel‐based RCA. The data represent the mean ± standard deviation of n = 2 technical replicates, with *n* = 3 replicates for the blank. Cross‐reactivity heatmap values were calculated from samples with on‐target AMB values nearest 1.5 on each dropout curve and represent the mean of duplicate measurements. (A) and illustrations in (D) were created with BioRender.

To inform our design of PRO‐MOSAIX, we first investigated potential sources of false positive signals in addition to cross‐reactive antibody‐antigen binding in MOSAIC‐based assays. As RCA and other DNA amplification methods can be prone to false positive signals arising from nonspecific amplification [24], we explored this possibility by performing spike‐in control experiments in which non‐target fluorescent dye‐encoded beads are added to beads with formed immunocomplex sandwiches labeled with circular DNA template, immediately prior to RCA (Figure 2A, top). We spiked in both antibody only‐coupled beads used in MOSAIC and antibody/DNA‐co‐coupled beads that would be used in PRO‐MOSAIX. Interestingly, we observed notable increases in the average molecules per bead (AMB) signals on the off‐target beads after RCA, when spiked into a bead mixture carrying high numbers of DNA template‐labeled immunocomplex sandwiches (Figure S1). This increase in false‐positive ON off‐target beads occurred for both antibody‐coupled and antibody/DNA‐co‐coupled beads. Such false‐positive signals may arise from nonspecific diffusion of DNA products across beads during RCA. While we were able to reduce these false positive signals by three‐ to five‐fold via tuned RCA conditions with shorter reaction times and addition of low concentrations of heparin and salt, the off‐target beads exhibited residual false positive signals from DNA product diffusion, with “cross‐reactivities” of 0.5‐0.6%, despite no exposure to analytes or detector antibodies (Figure S1).

We hypothesized that such nonspecific migration of DNA products can be inhibited by trapping the beads and DNA products in a reversibly formed hydrogel during RCA. In particular, we explored agarose gel encapsulation, as agarose gels can be readily melted by heating for bead retrieval and have been successfully used with RCA across various applications [25, 26, 27]. After non‐target beads were spiked into beads carrying DNA template‐labeled immunocomplexes, the beads were resuspended in RCA mixture, mixed with molten agarose, and immediately cooled on ice for gelation (Figure 2A, bottom). RCA was then carried out in the gel by incubating at 37°C, after which the gel was melted under optimized heating conditions to retrieve beads for washing, fluorescent probe labeling of RCA products, and flow cytometric counting of ON and OFF beads (Figure S3). Compared to our optimized solution‐based RCA, spatial separation of beads and entrapment of DNA products in an agarose gel successfully reduced false‐positive signals on off‐target beads while maintaining high DNA amplification efficiencies (Figures 2B and S2A). Increasing gel density up to 3% agarose decreased off‐target bead ON signals, presumably via increased inhibition of DNA product diffusion.

As gel volume was increased from 200 µL to 800 µL for the same number of beads, cross‐reactive signals from DNA product migration did not show significant differences, although on‐target signal‐to‐background ratios were slightly increased at larger gel volumes (Figures 2C and S2B). We therefore selected larger gel volumes for subsequent three‐plex assays. To minimize potential nonspecific binding of free DNA products during gel melting, we tested and optimized salt and heparin concentrations in the buffer that was added post‐RCA to the gel prior to melting and used for subsequent bead washing (Figure S4). These results support our hypothesis that a dense hydrogel matrix can reduce or inhibit any nonspecific diffusion of large DNA products across spatially separated beads. Importantly, this method retains the streamlined, solution‐based readout of MOSAIC, requires no additional instrumentation or microfluidics, and is scalable for parallel processing.

Having verified the feasibility of our gel entrapment strategy for mitigating nonspecific DNA product diffusion across beads during signal amplification, we compared the cross‐reactive signals of a full three‐plex MOSAIC assay with solution‐ versus gel‐based RCA. Human IL‐12p70, IL‐10, and IL‐6 were used as model cytokine analytes that play important roles in inflammation, cancer, and infectious diseases [28, 29, 30], with corresponding capture beads each encoded with a distinct AlexaFluor 488 dye intensity. Dropout curves, in which increasing concentrations of single protein analytes are measured with the multiplex assay, were performed to assess cross‐reactivity. Notably, gel entrapment during RCA significantly decreased false‐positive signals on off‐target beads at increasing on‐target analyte concentrations (Figure 2D). Quantification of these false‐positive signals at high concentrations of each analyte yielded largely lower cross‐reactivities across off‐target beads using gel‐ versus solution‐based RCA (up to 0.61% and 3.35%, respectively). Our gel entrapment approach yielded sub‐ to low femtomolar limits of detection, which were slightly higher than the corresponding solution‐based RCA format (Table 1 and Figure S5). This modest reduction in sensitivity may be attributed to lower bead recoveries from the gel, thereby decreasing the number of counted events and measurement precision at low concentrations. However, these detection limits remain comparable in range to other digital immunoassay platforms [6, 10] and at least one to two orders of magnitude lower than existing cytokine assays that have been clinically approved or are used in Clinical Laboratory Improvement Amendments (CLIA)‐certified laboratories. As these cytokines typically exist at low to mid‐femtomolar concentrations or below in human plasma and at picomolar concentrations in various disease states, the sensitivity and dynamic range (about three orders of magnitude) of the multiplex MOSAIC assay, with both gel and solution RCA, are clinically relevant, potentially improving detectability and measurement resolution while allowing sample conservation.

**TABLE 1:** Limit of detection (LOD) and lower limit of quantification (LLOQ) values of three‐plex MOSAIC assays with solution‐ and gel‐based RCA^a^. Representative clinically relevant concentration and assay ranges are shown for each analyte.

			MOSAIC—Solution RCA		MOSAIC – Gel RCA	
Analyte	Estimated concentration range in human plasma	Clinical or CLIA‐certified assay ranges^b^	LOD (aM)	LLOQ (aM)	LOD (aM)	LLOQ (aM)
IL‐12p70	≤ low fM to sub‐pM [31]	mid‐fM to mid‐pM	20.6 ± 8.2 [11.3 − 26.2]	96.2 ± 4.5 [92.7 − 101.2]	190.4 ± 225.0 [51.5 − 450.0]	545.1 ± 605.2 [169.4 − 1243.3]
IL‐10	≤ mid‐fM to mid‐pM [31, 32, 33]	mid‐fM to mid‐pM	87.1 ± 75.6 [19.7 − 168.9]	442.7 ± 148.7 [278.6 − 568.6]	507.8 ± 168.5 [326.0 − 658.8]	1597.2 ± 505.1 [1029.8 − 1997.8]
IL‐6	≤ mid‐fM to high pM [31, 32]	mid‐fM to high pM	593.0 ± 175.8 [446.6 − 788.0]	4298.9 ± 2761.7 [2057.0 − 7384.0]	1109.4 ± 689.7 [336.0 − 1660.4]	3868.4 ± 1998.1 [1580.3 − 5269.4]

^a^ The LOD and LLOQ values were calculated as the concentrations corresponding to three and 10 standard deviations, respectively, above the background AMB. Values represent the mean ± standard deviation [range] of at least two independent calibration curves performed on different days.

^b^ General assay ranges are estimated from clinically approved (Roche Elecsys IL‐6 [34]) or representative immunoassays performed as laboratory developed tests in Clinical Laboratory Improvement Amendments (CLIA)‐certified laboratories (e.g., Eurofins Viracor Meso Scale Discovery multiplex array).

### 2.2 Incorporation of Proximity‐Based Barcoding for Accurate Multiplexed Single‐Molecule Protein Detection

**FIGURE 3:**
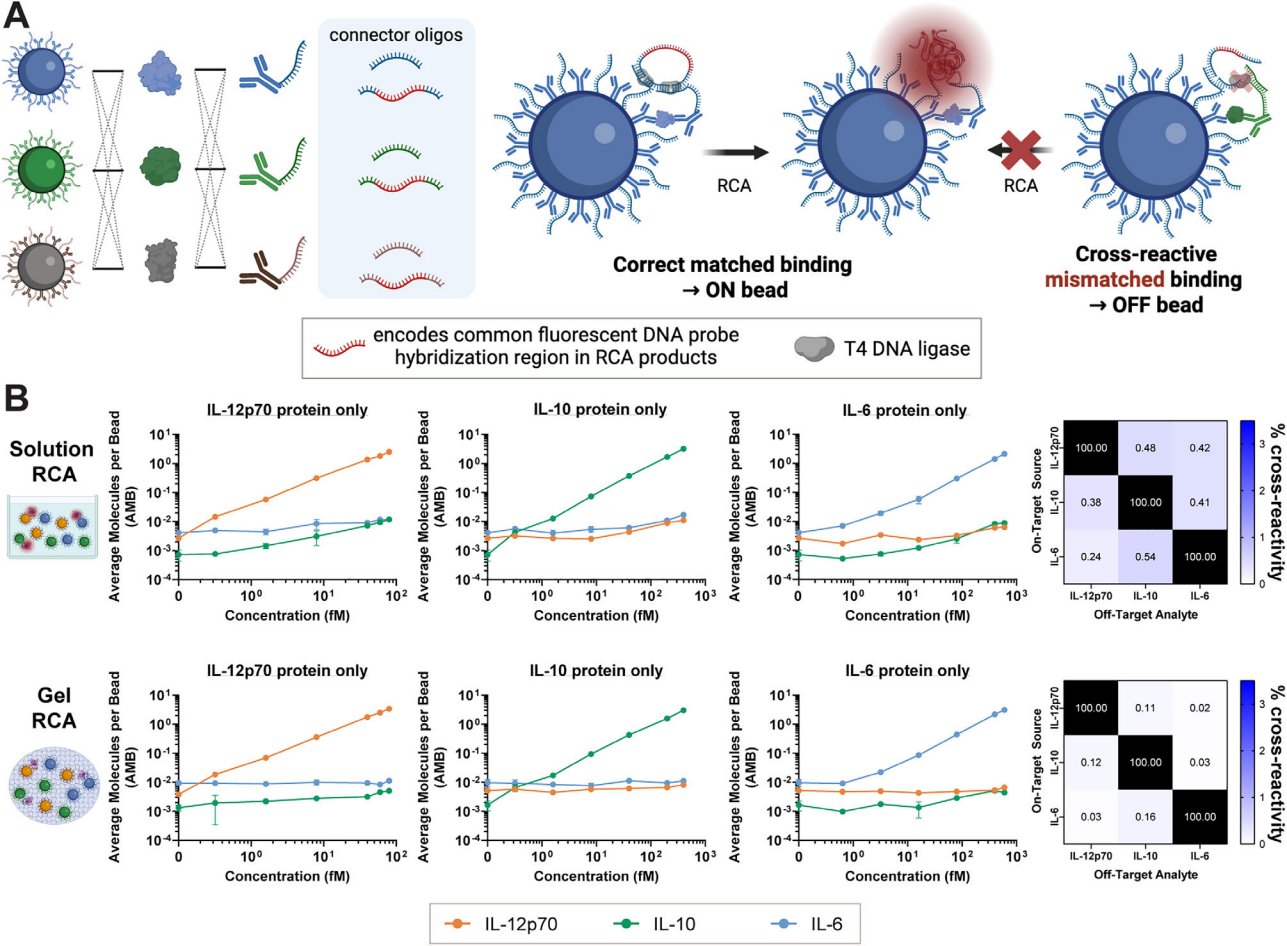
Proximity‐based barcoding strategy in PRO‐MOSAIX for multiplexed single‐molecule protein detection. (A) Schematic of proximity ligation‐based barcoding. Dye‐encoded beads are co‐coupled with capture antibody and proximity DNA barcode, while detector antibodies are conjugated with a paired proximity DNA barcode. Once single immunocomplex sandwiches are formed, connector oligos are hybridized and ligated into a circular template. Connector oligos comprise hybridization sequences specific for each proximity barcode pair and a common sequence (red) for fluorescent probe hybridization to RCA products. RCA is then carried out, generating a localized fluorescent “ON” signal on each bead carrying a correct immunocomplex sandwich. Signal is produced only when matched capture–detector pairs bind the same target molecule, enabling accurate high‐order multiplexed detection through proximity barcoding. (B) Protein dropout curves across increasing concentrations of individual target proteins to evaluate cross‐reactivities in a three‐plex PRO‐MOSAIX assay with solution‐ and gel‐based RCA. The data represent the mean ± standard deviation of *n* = 2 technical replicates, with *n* = 3 replicates for the blank. Cross‐reactivity heatmap values were calculated from samples with on‐target AMB values nearest 1.5 on each dropout curve and represent the mean of duplicate measurements. Cross‐reactivity (%) is defined as the off‐target/on‐target AMB ratio after background subtraction. (A) and illustrations in (B) were created with BioRender.

We next introduced proximity‐based barcoding into the multiplex assay workflow to develop the PRO‐MOSAIX platform. For each of the analytes IL‐12p70, IL‐10, and IL‐6, dye‐encoded beads were co‐coupled with capture antibody and a proximity oligo barcode, and detector antibody was conjugated with a paired proximity oligo barcode (Figure 3A and Figure S6). After single immunocomplex sandwiches are formed using a high excess number of beads over target molecules for each analyte, the capture and detector proximity DNA barcodes template the circularization and ligation of added connector DNA oligos. Connector oligos comprise hybridization sequences specific to each proximity barcode oligo pair and a common sequence for ATTO647N‐DNA probe hybridization after RCA, thus requiring only one fluorescence channel to be used for “ON” bead signal readout. In parallel, the versatility of ratiometric fluorescent dye encoding of beads [35, 36] enables the generation of a large array of capture beads that are distinguishable by flow cytometry.

Integration of proximity barcoding maintained mid‐aM to low fM sensitivities, with comparable detection limits as MOSAIC, for both solution‐ and gel‐based RCA (Table 2 and Figure S7). While proximity barcoding reduced relative cross‐reactive signals in solution‐based RCA format compared to the non‐barcoded three‐plex MOSAIC assay, dropout curves still exhibited increasing false positive signals at high single protein concentrations (Figure 3B). When we further incorporated gel entrapment during RCA, false positive signals were largely eliminated in the resultant three‐plex PRO‐MOSAIX assay, with minimal cross‐reactivities (up to 0.16%). This trend was consistent with our previous observation of reduced cross‐reactive signals in the three‐plex MOSAIC assay with gel‐based RCA, further supporting the efficacy of our gel entrapment strategy in mitigating false positive signals arising during DNA amplification. Notably, PRO‐MOSAIX outperformed the gel‐based non‐barcoded MOSAIC assay in minimizing cross‐reactivity, suggesting that proximity‐dependent signal generation in our DNA‐paired barcoding strategy can indeed reduce false positive ON bead signals from mismatched antibody‐antigen binding.

**TABLE 2:** LOD and LLOQ values of three‐plex PRO‐MOSAIX assays with solution‐ and gel‐based RCA.^a^

	PRO‐MOSAIX – Solution RCA		PRO‐MOSAIX – Gel RCA	
Analyte	LOD (aM)	LLOQ (aM)	LOD (aM)	LLOQ (aM)
IL‐12p70	25.2 ± 9.3 [18.7 − 31.8]	109.1 ± 8.0 [103.4 − 114.8]	46.9 ± 36.6 [17.1 − 109.9]	160.9 ± 109.2 [49.6 − 182.5]
IL‐10	167.3 ± 101.9 [95.3 − 239.4]	573.3 ± 240.1 [403.6 − 743.1]	295.5 ± 319.1 [45.8 − 839.2]	1095.8 ± 934.9 [181.1 − 2487.3]
IL‐6	518.7 ± 311.8 [298.2 − 739.1]	1741.1 ± 991.9 [1,039.7 − 2442.4]	1065.2 ± 406.2 [488.1 − 1558.7]	3776.2 ± 1215.6 [1660.3 − 4681.0]

^a^ The LOD and LLOQ values were calculated as the concentrations corresponding to three and 10 standard deviations, respectively, above the background AMB. Values represent the mean ± standard deviation [range] of at least two independent calibration curves performed on different days.

**FIGURE 4:**
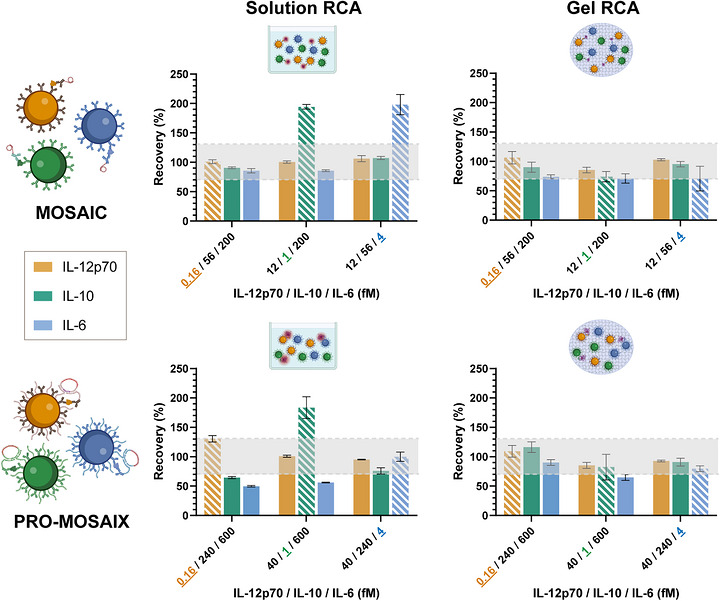
Measurement accuracies of three‐plex MOSAIC and PRO‐MOSAIX assays. Mixtures of recombinant proteins at varying high and low concentrations were measured with three‐plex MOSAIC and PRO‐MOSAIX assays, with solution‐ or gel‐based RCA. Recoveries were calculated as measured concentration divided by actual concentration of each analyte, with the acceptable 70%–130% range highlighted in gray. Low‐concentration analytes within each mixture are denoted by striped bars, with low concentrations selected near the assay LLOQ. High analyte concentrations were selected near the upper end of the assay dynamic range. The data represent the mean ± standard deviation of *n* = 3 technical replicates. Illustrations were created with BioRender.

To further validate the accuracy of PRO‐MOSAIX, we measured mixtures of varying high and low concentrations of the three protein analytes in the multiplex assay (Figure 4). Measurement accuracies were assessed by comparing the measured and actual concentrations of each protein and calculating percent recovery. For each assay, the low concentration of each analyte within a mixture was selected to be slightly above the LLOQ, and the high concentration of each analyte was selected near the upper end of the corresponding calibration curve. Consistent with the high cross‐reactivities observed in our dropout experiments, the three‐plex MOSAIC assay with solution‐based RCA yielded the least accurate measurements, with recoveries of approximately 200% for multiple low concentration analytes in the presence of high concentrations of other analytes. Incorporation of proximity barcoding while retaining the solution‐based RCA format improved measurement accuracies for low concentration analytes, but still resulted in overestimated measurements in one of the mixtures. When we further integrated our gel entrapment strategy, the resultant PRO‐MOSAIX assay achieved high measurement accuracies, with 70%–130% recoveries across all low concentration and nearly all high concentration analytes. For the three‐plex MOSAIC assay, the gel entrapment strategy was also able to eliminate overestimation of low concentration analytes. These results suggest that much of the false positive signals in the three‐plex assay arise during DNA signal amplification and are mitigated by our gel entrapment strategy during RCA. While cross‐reactive antibody‐antigen interactions are fewer in a three‐plex assay compared to high‐order multiplex assays, PRO‐MOSAIX together with gel‐based RCA yielded the most consistent reduction in cross‐reactivities along with high measurement accuracies.

Importantly, the PRO‐MOSAIX assay demonstrated high precision with intra‐assay coefficients of variation (CVs) consistently below 20% at concentrations above the LLOQ (Table S1). Measurements of the same control samples across independent runs also yielded CVs below 20%, confirming high inter‐day reproducibility. We further assessed batch reproducibility and reagent stability, showing that reagents stored from weeks to over two years yielded comparable assay sensitivities with similar LOD and LLOQ values across all three analytes (Table S2). Together, these results demonstrate the robust and reproducible performance of the PRO‐MOSAIX platform.

### 2.3 Development of an Ultrasensitive 15‐plex PRO‐MOSAIX Assay

To further assess the ability of proximity barcoding to minimize cross‐reactivities from increasing mismatched antibody‐antigen interactions, we next expanded PRO‐MOSAIX to a larger multiplex panel. While up to eight‐plex digital ELISA assays have been demonstrated to date, the absence of cross‐reactivity control mechanisms has limited multiplex accuracy and order, while fluorescence spectral overlap in signal readout has limited barcoding capacity in cross‐reactivity mitigation strategies [9, 11, 37]. As PRO‐MOSAIX allows the same fluorescence channel to be used for ON signals across all targets while providing a signal selection filter for matched affinity reagent pairs, we explored its high‐order multiplexing capabilities in a proof‐of‐principle ultrasensitive 15‐plex assay, representing a three‐fold increase in barcoded multiplexing in digital ELISA [11]. To generate an array of 15 distinguishable capture beads, we labeled carboxylated paramagnetic beads with unique ratios of up to three fluorescent dyes (Figure S8 and Table S3). Each encoded bead was subsequently co‐coupled with a specific capture antibody and proximity DNA barcode. As target analytes, we chose both low‐ and medium‐abundance blood biomarkers to test the versatility in dynamic range of PRO‐MOSAIX. Our panel included an expanded set of cytokines that regulate immune and inflammatory responses in diverse diseases [38, 39, 40, 41], as well as the tumor biomarkers CA125 and HE4 [42, 43].

With over three‐fold higher total number of beads in the 15‐plex assay (20,000 beads per target) compared to our previous three‐plex assay (30,000 beads per target), we tested increased gel volumes for RCA. Bead spike‐in experiments during RCA showed similar cumulative cross‐reactivities across off‐target beads from DNA product diffusion as gel volume was increased beyond the original 800 µL used for the three‐plex assay (Figure S9A). However, we noted a slightly increased signal‐to‐background for certain on‐target beads when gel volume was increased up to 1600 µL (Figure S9B). We therefore selected higher gel volumes for our next 15‐plex experiments. In addition, due to the increased number of DNA oligos in the 15‐plex PRO‐MOSAIX assay, we used a thermotolerant DNA ligase, Hi‐T4, at elevated ligation temperature to minimize potential false‐positive signals from nonspecific hybridization and ligation of mismatched DNA barcodes. Performing the ligation step at an increased temperature of 45 °C with Hi‐T4 DNA ligase, together with splitting the DNA hybridization and ligation into separate steps, maintained mostly similar on‐target signal‐to‐background ratios across multiple analytes (Figure S10A).

**FIGURE 5:**
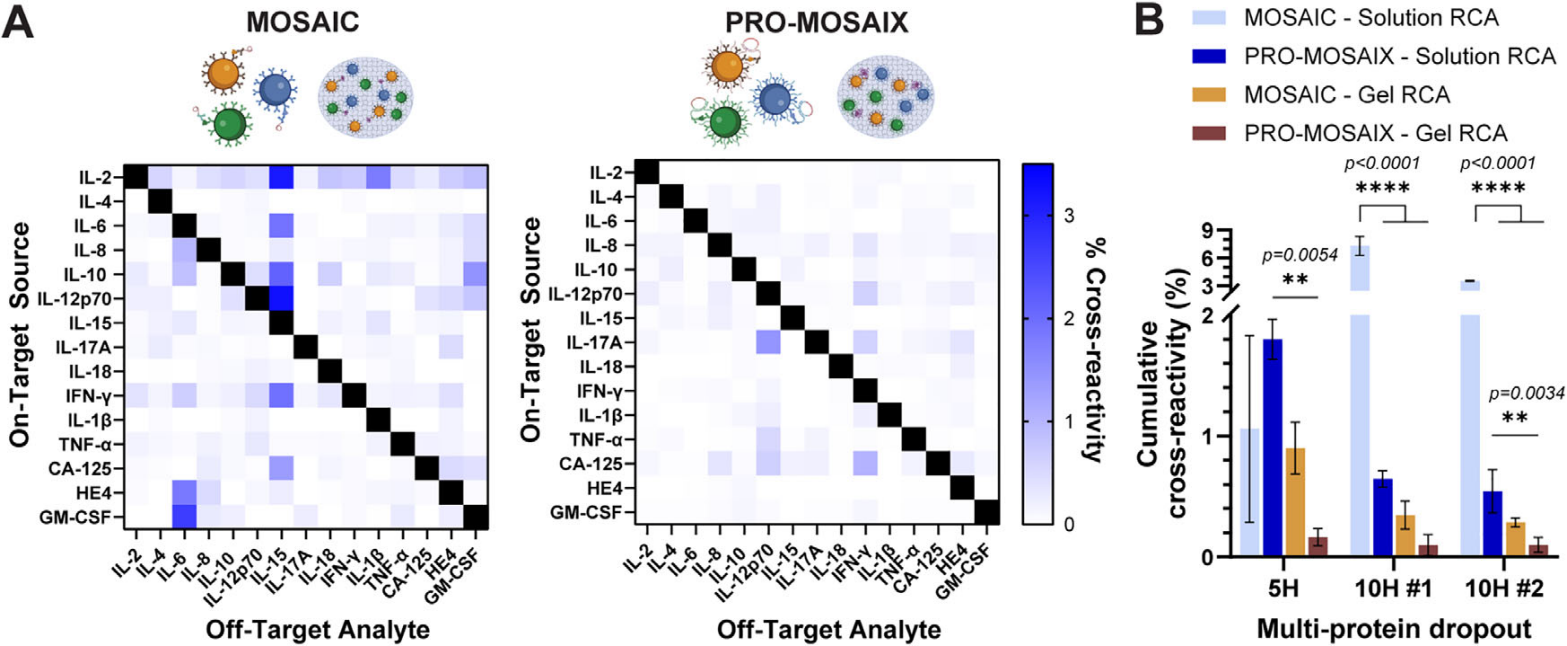
Cross‐reactivity assessment in 15‐plex MOSAIC and PRO‐MOSAIX assays using single‐ and multi‐protein dropout mixtures. (A) Heatmaps of percent cross‐reactivity in 15‐plex assays with gel‐based RCA, in measurements of single high‐concentration proteins. A gel volume of 2400 µL was used for RCA. Percent cross‐reactivity is defined as the off‐target/on‐target AMB ratio after background subtraction. The data represent the mean of duplicate measurements. (B) Cumulative cross‐reactivities in 15‐plex MOSAIC and PRO‐MOSAIX assays in measurements of multi‐protein dropout mixtures containing pooled high concentrations of five or 10 proteins. A gel volume of 1600 µL was used for RCA. Cumulative cross‐reactivity is defined as the sum of off‐target AMB values divided by the sum of on‐target AMB values after background subtraction. The data represent the mean ± standard deviation of *n* = 3 technical replicates and were analyzed by one‐way ANOVA with Tukey's post‐hoc test for multiple comparisons. **p<0.01; ****p<0.0001. Mixture components are listed in Table S4. Illustrations in (A) were created with BioRender.

To evaluate cross‐reactivity, we measured high‐concentration single protein samples with the 15‐plex PRO‐MOSAIX and corresponding MOSAIC assays. Consistent with our three‐plex assay results, PRO‐MOSAIX integrated with gel‐based RCA yielded minimal cross‐reactivities (Figure 5A). In comparison, the corresponding 15‐plex MOSAIC assay exhibited higher cross‐reactivities of up to over 3%, suggesting that proximity‐based barcoding effectively mitigates false positive ON signals from increasing combinatorial cross‐reactive antibody‐antigen interactions with higher‐order multiplexing. Elevation of ligation temperature with Hi‐T4 DNA ligase slightly lowered overall cross‐reactive signals in PRO‐MOSAIX, suggesting alleviation of potential mismatch ligation events (Figure S10B). For both MOSAIC and PRO‐MOSAIX, signal amplification in solution format yielded higher cross‐reactivities compared to gel format, similar to our observations in the three‐plex assays (Figure S11).

The 15‐plex assays were then further challenged with multi‐protein dropout mixtures comprising pooled high concentrations of five or 10 proteins (Figure 5B and Table S4). With solution‐based RCA, proximity barcoding substantially reduced, but did not fully eliminate cumulative cross‐reactivities across off‐target beads in mixtures comprising 10 high‐concentration proteins. Similar to our single‐protein dropout results, incorporating gel‐based RCA generally decreased cumulative cross‐reactivity for both MOSAIC and PRO‐MOSAIX across different mixtures. Notably, combining proximity barcoding and gel‐based RCA consistently yielded the lowest cumulative cross‐reactivities across all mixtures. These trends were maintained across different gel volumes, indicating robust suppression of cross‐reactivity with our integrated approach (Figures S12, S13).

We next assessed the analytical sensitivities of the 15‐plex PRO‐MOSAIX and MOSAIC assays. Notably, we observed significantly higher backgrounds and reduced dynamic ranges across multiple analytes in the 15‐plex MOSAIC assay compared to PRO‐MOSAIX (Figures S14‐S16). Several analytes with particularly elevated backgrounds in MOSAIC, including IL‐6, IL‐15, and GM‐CSF, exhibited less than two orders of magnitude in dynamic range and an order of magnitude lower sensitivity compared to PRO‐MOSAIX (Figure S16 and Table S5). Such a difference can be attributed to the cumulative nonspecific binding of all detector antibodies to each capture bead in multiplex MOSAIC. As every nonspecifically bound detector antibody can generate an ON signal, the background for each analyte increases with increasing multiplex panel size, which can reduce dynamic range and sensitivity. These elevated backgrounds and reduced dynamic ranges occurred in MOSAIC for solution‐based RCA as well (Figure S17 and Table S6). In contrast, the proximity barcoding strategy of PRO‐MOSAIX restricts the background for each analyte in the multiplex assay to the nonspecific binding of only the corresponding detector antibody to the matched capture bead. With lower backgrounds and broader dynamic ranges, the 15‐plex PRO‐MOSAIX assay achieved sub‐ to low femtomolar limits of detection across most analytes.

### 2.4 Validation of 15‐plex PRO‐MOSAIX Assay in Human Plasma

Finally, we evaluated the measurement accuracies of the 15‐plex PRO‐MOSAIX assay and its analytical performance in human plasma, using the gel RCA‐integrated format. As the protein analytes span different concentration ranges in plasma, the assay dynamic range for each analyte can be readily tuned via detector antibody concentration and assay bead number [9]. We therefore decreased the detector antibody concentration for HE4, which can exist at mid‐picomolar to low nanomolar concentrations in plasma, with particularly high levels in ovarian cancer patients [44, 45]. The resultant 15‐plex PRO‐MOSAIX assay demonstrated sub‐ to high femtomolar detection limits, with a cumulative dynamic range of over six orders of magnitude across the 15 analytes (Figure S18 and Table S7).

**FIGURE 6:**
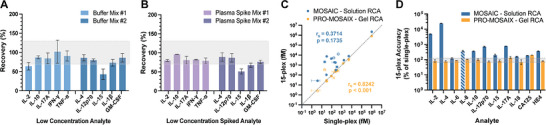
Measurement accuracies of 15‐plex PRO‐MOSAIX assay in protein mixtures and human plasma. (A,B) Recovery rates of 15‐plex PRO‐MOSAIX assay for low concentration analytes in recombinant protein mixtures in buffer (A) and spiked into 12‐fold diluted pooled human plasma mixtures (B). Each mixture in buffer or spiked into plasma comprised five low and 10 high concentration protein analytes. Low concentrations were selected based on the assay LLOQ or standard deviation of the measured endogenous concentration in plasma; high concentrations were selected based on the upper end of the assay dynamic range. A gel volume of 1600 µL was used for RCA. Recoveries were calculated as the ratio of measured to actual analyte or spike concentrations, with the acceptable 70%–130% range shaded in gray. Analyte concentrations are listed in Table S8. (C) Measured individual analyte concentrations in 12‐fold diluted human pooled plasma using 15‐plex MOSAIC (solution RCA) and PRO‐MOSAIX (gel RCA, with a gel volume of 800 µL) assays, compared to benchmark single‐plex MOSAIC assays. Concentrations shown represent the endogenous plasma concentrations calculated from measured concentration values at 12‐fold dilution. The gray dashed line denotes perfect positive linear correlation between the multiplex and single‐plex assays. The Spearman correlation coefficients (r_s_) were 0.3714 for the 15‐plex MOSAIC (solution RCA) assay and 0.8242 for the PRO‐MOSAIX (gel RCA) assay, with two‐tailed p values of 0.1735 and 0.0009, respectively. Samples below the 15‐plex assay LOD were assigned the LOD value. Symbols denote detectability status as follows: filled circles, detected in both assays; open circles, detected in 15‐plex only; open triangles, detected in single‐plex only; and crosses (×), undetectable in both assays. (D) Accuracy of endogenous plasma protein quantification using the 15‐plex MOSAIC (solution RCA) or PRO‐MOSAIX (gel RCA, with a gel volume of 800 µL) assay compared to benchmark single‐plex MOSAIC assays in 12‐fold diluted pooled human plasma. Accuracy of 15‐plex measured concentrations is expressed as the percentage of the measured concentration with a single‐plex MOSAIC assay, with an acceptable 70%–130% range shaded in gray. Measured IL‐8 concentration using the 15‐plex MOSAIC assay exceeded the assay dynamic range and the corresponding accuracy is denoted as a striped bar and determined at the highest IL‐8 calibrator concentration. Accuracies are listed in Table S11. All data represent the mean ± standard deviation of *n* = 2–3 technical replicates.

In mixed panels containing high‐ and low‐concentration proteins, PRO‐MOSAIX achieved high accuracies across nearly all low‐concentration analytes in two distinct mixtures, yielding 70%–130% recoveries even in the presence of 10 high‐concentration off‐target proteins (Figures 6A, S19A, and Table S8). When such mixtures were spiked into 12‐fold diluted human pooled plasma, the 15‐plex PRO‐MOSAIX assay maintained high measurement accuracies for both low‐ and high‐concentration spiked analytes (Figures 6B and S19B). We further verified robust recoveries in mixtures containing all 15 recombinant proteins spiked at medium and high concentrations into diluted plasma (Table S9). Thus, the integrated PRO‐MOSAIX platform achieves consistent measurement accuracies across low‐ and high‐concentration analytes in various mixtures in both buffer and plasma.

To further validate the accuracy of the 15‐plex PRO‐MOSAIX in plasma, we compared the measured endogenous concentrations of several proteins in pooled human plasma with those obtained from corresponding single‐plex MOSAIC assays (Figure 6C,D). We utilized lower gel RCA volumes of 800 µL for these experiments to reduce assay costs, verifying that analytical sensitivities and robust cross‐reactivity mitigation were maintained (Figures S13, S20; and Table S10). We further observed about two‐fold higher bead recoveries of 50%–70% from gel RCA in the 15‐plex assay compared to the three‐plex assay, likely due to the increased number of starting beads (Figure S21). Notably, PRO‐MOSAIX measurements exhibited much stronger agreement than the 15‐plex MOSAIC measurements with the single‐plex benchmarks across analytes spanning femtomolar to picomolar concentrations (Spearman correlation coefficients of 0.8242 and 0.3714 for PRO‐MOSAIX and MOSAIC, respectively). This discrepancy was largely driven by overestimated concentrations of multiple analytes in the multiplex MOSAIC assay, with several overestimations by one to two orders of magnitude (Figure 6D and Table S11). Such overestimation is consistent with the expected accumulation of nonspecific detector antibody binding in larger multiplex MOSAIC panels, which is amplified in complex biofluids containing interfering matrix components that increase nonspecific binding. The paired proximity barcoding mechanism in PRO‐MOSAIX bypasses this cumulative nonspecific binding problem, thereby minimizing inflated background signals and increasing measurement accuracies in biofluids. While such concentration overestimation by the 15‐plex MOSAIC assay was less evident in several other plasma samples, the inconsistent measurement accuracies among different plasma samples indicate that non‐barcoded multiplex MOSAIC is not robust to various matrix interference effects and nonspecific binding across individual biofluids (Figure S22). In contrast, PRO‐MOSAIX demonstrated consistent accuracies across all measured plasma samples. Collectively, these results demonstrate that the PRO‐MOSAIX platform can simultaneously quantify endogenous proteins at femtomolar concentrations in human plasma with high accuracies comparable to single‐plex MOSAIC assays.

Finally, to assess platform scalability for future work, we compared overall assay costs and times of PRO‐MOSAIX with those of commercial digital ELISA (Single‐molecule arrays). The 15‐plex PRO‐MOSAIX assay can be performed at about $2 or $5 per sample with solution or gel RCA, respectively, achieving three‐ to seven‐fold lower costs compared to commercial multiplex digital ELISA kits while also utilizing common laboratory instrumentation (Table S12). While the 15‐plex PRO‐MOSAIX assay with gel RCA currently requires five hours per sample from assay start to signal readout, using solution RCA significantly shortens assay time and increases sample throughput. As our data indicate that proximity barcoding alone already significantly reduces cross‐reactivities in challenging multi‐high concentration protein mixtures (Figures 5B and S12, S13), PRO‐MOSAIX with solution RCA can enable accurate ultrasensitive multiplex measurements in many applications while allowing shorter assay times and a high‐throughput, fully plate‐based workflow. We evaluated the feasibility of further reducing assay times for the 15‐plex PRO‐MOSAIX assay using solution RCA, by shortening capture time and combining the hybridization and ligation steps to a total of two hours per sample (Table S13). The resultant assays maintained similar sensitivities across most analytes compared to the longer PRO‐MOSAIX assays with solution or gel RCA. These sensitivities were also comparable to or within one order of magnitude of those of commercial 4‐plex digital ELISA kits. Such assay parameter optimization can also be applied to PRO‐MOSAIX assays that use gel RCA to reduce assay time. Continued assay‐specific optimization and liquid handling automation present future opportunities for enhancing throughput while opening the door to high‐order multiplexing in digital ELISA.

## 3 Conclusion

Proteins play critical roles in nearly all biological and pathological processes, but their vast informational and clinical potential remains unrealized due to technological gaps in protein detection and the low abundances of many proteins. While the exceptional sensitivities of digital ELISA methods have expanded the window of accessible proteins, limited multiplexing capacity and the requirement for specialized instrumentation remain key challenges. We have developed a modular digital ELISA platform, PRO‐MOSAIX, that bridges the gap between ultrasensitive protein detection and robust, high‐fidelity multiplexing capabilities, using common laboratory equipment. By incorporating proximity ligation with paired DNA barcodes on matched affinity reagents, our platform eliminates false positive signals from cross‐reactive binding interactions. Such a strategy enables a single fluorescence channel to be used for ON signals across all capture beads, greatly increasing multiplexing capacity while ensuring accuracy. We further enhance measurement accuracies by addressing nonspecific DNA product diffusion across beads during signal amplification, using a reversibly formed gel to physically trap such DNA products.

Our integrated platform achieves mid‐attomolar to low femtomolar sensitivities across multiple analytes, demonstrating accurate measurements across various high‐ and low‐concentration analyte mixtures and in plasma. The dynamic range for each analyte in the multiplex assay can be independently tuned via detector antibody concentration and capture bead number, as has been shown in MOSAIC and other digital ELISA platforms [9, 11, 46]. Such tunability allows broad coverage across low and high‐abundance proteins in a single measurement at a given dilution factor, with our 15‐plex PRO‐MOSAIX assay achieving a cumulative dynamic range of over six orders of magnitude across all analytes. Dynamic range can be further expanded in future applications by extending AMB signals beyond the digital regime to incorporate analog values, similar to existing digital ELISA platforms [47]. Furthermore, our proximity barcoding strategy markedly reduces background and yields broader assay dynamic ranges along with improved analytical sensitivities compared to conventional multiplex MOSAIC and other digital immunoassays, particularly as multiplex order increases. In complex biofluids, the cumulative nonspecific binding of detector affinity reagents in conventional multiplex digital immunoassays can be exacerbated by matrix components, further compromising measurement accuracies. Such potential interference is mitigated in PRO‐MOSAIX, which confines background to the nonspecific binding of only the cognate detector affinity reagent. The solution‐based flow cytometric readout further bypasses limitations on dynamic range and multiplex order faced by digital immunoassay platforms that rely on physical compartmentalization. Altogether, PRO‐MOSAIX mitigates challenges of cross‐reactivities and cumulative nonspecific binding that limit high‐order multiplexing in ultrasensitive protein detection.

There remain several limitations in the current work and avenues for future investigation. Our proof‐of‐principle 15‐plex PRO‐MOSAIX assay represents a three‐fold increase in barcoding capacity over existing cross‐reactivity mitigation approaches in digital ELISA [11, 13] and is the highest multiplex order in digital ELISA to date, overcoming multiplexing fidelity and spectral overlap limitations in ultrasensitive single‐molecule protein detection. While its current multiplexing scale remains lower than those of several commercial multiplex protein detection platforms and proteomics tools, PRO‐MOSAIX can be readily expanded to much higher multiplex orders in future work due to the versatility of DNA barcoding. As ratiometric fluorescent dye labeling of beads and varying bead sizes can enable hundreds of distinguishable beads [35, 36, 48], we expect that optimization and expansion of the fluorescent dye labeling used in this work, along with exploration of additional dye conjugation methods, can yield similarly large arrays.

In addition, while our 15‐plex PRO‐MOSAIX assay achieved sub‐ to low femtomolar detection limits, further improvements in sensitivity are required for robust detection of lower‐abundance analytes at attomolar or below concentrations. Toward this front, we anticipate that assay bead recoveries in PRO‐MOSAIX assays utilizing gel RCA can be increased upon further optimization of gel dissolution and exploration of additional gel materials. As more beads are counted, increased sampling efficiency reduces Poisson counting error and enables assay bead number tuning for maximizing signal‐to‐background ratios while maintaining measurement precision. Furthermore, our data suggest that the increasing total bead number with increasing multiplex order improves bead recoveries, with the 15‐plex PRO‐MOSAIX assay achieving 50%–70% bead recoveries from gel RCA. In conjunction, screening and development of additional affinity reagents can be performed to enhance sensitivities as required for rare analytes. Importantly, proximity barcoding removes the requirement for extensive validation of affinity reagents in conventional multiplex assays for cross‐reactivities, considerably increasing flexibility in affinity reagent screening and assembly of large multiplex panels.

A key translational bottleneck of our current PRO‐MOSAIX platform is the decreased workflow scalability when incorporating gel‐based RCA. While our data show that proximity barcoding with solution RCA already significantly reduces cross‐reactivities in challenging protein mixtures, gel RCA will further increase measurement accuracies in applications where multiple panel analytes have endogenous concentrations near the upper end of the calibration curve, the regime where nonspecific DNA diffusion during RCA contributes most to cross‐reactive signals. Although the gel‐based RCA method is integrated with an automated flow cytometric readout, the use of tubes for gel formation and dissolution significantly increases workflow complexity and reduces throughput compared to solution‐based RCA in multi‐well plates. Scalability can be increased in future work using deep‐well plates or adjustable multichannel pipettes in appropriate setups. The sample processing steps of PRO‐MOSAIX are also amenable to semi‐ or full automation with robotic liquid handlers. In conjunction, future work will explore additional reversible hydrogel materials to further tune gel mesh size and dissolution steps, to enable small gel volumes for high‐throughput 96‐ or 384‐well plate processing while ensuring robust inhibition of DNA product diffusion during signal amplification. Alternative hydrogel materials with bio reducible properties or pH‐labile bonds can also bypass the need for heating steps in gel dissolution. However, for applications where most panel analytes have endogenous concentrations in the lower half of the calibration curve, PRO‐MOSAIX with solution RCA enables sufficient cross‐reactivity reduction using 96‐well plate‐based processing across all assay steps. In such cases, this integrated workflow closely mirrors those of prior high‐throughput MOSAIC platforms [9, 11], thus presenting a more feasible translational path.

Finally, PRO‐MOSAIX utilizes standard laboratory instrumentation, including benchtop flow cytometry, which is widely used across research and clinical labs. While such a workflow significantly reduces infrastructural barriers compared to commercial digital ELISA and many multiplex protein detection platforms, integration into a point‐of‐care amenable format remains a future avenue for exploration. A conventional fluorescence imaging readout has previously been used for a digital ELISA method utilizing RCA amplification, based on a dried dropcast film of beads [8], thus supporting the feasibility of imaging readouts in PRO‐MOSAIX. Portable fluorescence imagers [49, 50] and integrated bead‐based microfluidic chips [51, 52, 53] can potentially be adapted to translate the readout and processing steps of PRO‐MOSAIX for use in resource‐limited settings.

In summary, PRO‐MOSAIX expands high‐fidelity multiplexing capabilities in single‐molecule protein detection while utilizing common laboratory infrastructure with a high‐throughput signal readout. The proximity‐dependent pairing of affinity reagents not only improves the analytical sensitivities and accuracies of multiplex digital immunoassays in biofluids, but also simplifies signal readout to a single fluorescence channel. PRO‐MOSAIX, thus provides a scalable path toward substantially expanding multiplex panel sizes without compromising assay performance. Its modular design, femtomolar and below sensitivities, and accessible workflow can potentially accelerate biomarker signature discovery, enable sample conservation, and maximize biological insight across basic and clinical applications.

## Author Contributions

**Connie Wu**: conceptualization, investigation, funding acquisition, writing – original draft, writing – review and editing, methodology, formal analysis, project administration, resources, supervision, and visualization. **Chi‐Chia Wang**: investigation, writing – original draft, writing – review and editing, methodology, formal analysis, and visualization. **Emily Dorsey**: investigation, writing – review and editing, validation, and formal analysis.

## Conflicts of Interest

C. Wu is an inventor on a patent application related to the MOSAIC platform used in part of this work and licensed to Quanterix Corporation. The University of Michigan has filed a provisional patent application on the technology reported in this work.

[Correction added on 1 May 2026, after first online publication: The Conflict of Interest was updated.]

## Supporting information




**Supporting File**: The authors have cited additional references within the Supporting Information.

## Data Availability

The data that support the findings of this study are available from the corresponding author upon reasonable request.

## References

[1] R. Aebersold , J. N. Agar , I. J. Amster , et al., “How Many Human Proteoforms are There?,” Nature Chemical Biology 14 (2018): 206–214, 10.1038/nchembio.2576. 29443976 PMC5837046

[2] E. W. Deutsch , G. S. Omenn , Z. Sun , et al., “Advances and Utility of the Human Plasma Proteome,” Journal of Proteome Research 20 (2021): 5241–5263, 10.1021/acs.jproteome.1c00657. 34672606 PMC9469506

[3] R. C. Fitzgerald , A. C. Antoniou , L. Fruk , and N. Rosenfeld , “The Future of Early Cancer Detection,” Nature Medicine 28 (2022): 666–677, 10.1038/s41591-022-01746-x. 35440720

[4] O. Hansson , “Biomarkers for Neurodegenerative Diseases,” Nature Medicine 27 (2021): 954–963, 10.1038/s41591-021-01382-x. 34083813

[5] P. E. Geyer , D. Hornburg , M. Pernemalm , et al., “The Circulating Proteome─Technological Developments, Current Challenges, and Future Trends,” Journal of Proteome Research 23 (2024): 5279–5295, 10.1021/acs.jproteome.4c00586. 39479990 PMC11629384

[6] D. M. Rissin , C. W. Kan , T. G. Campbell , et al., “Single‐Molecule Enzyme‐linked Immunosorbent Assay Detects Serum Proteins at Subfemtomolar Concentrations,” Nature Biotechnology 28 (2010): 595–599, 10.1038/nbt.1641. PMC2919230 20495550

[7] L. Cohen , N. Cui , Y. Cai , et al., “Single Molecule Protein Detection With Attomolar Sensitivity Using Droplet Digital Enzyme‐Linked Immunosorbent Assay,” ACS Nano 14 (2020): 9491–9501, 10.1021/acsnano.0c02378. 32589401

[8] C. Wu , P. M. Garden , and D. R. Walt , “Ultrasensitive Detection of Attomolar Protein Concentrations by Dropcast Single Molecule Assays,” Journal of the American Chemical Society 142 (2020): 12314–12323, 10.1021/jacs.0c04331. 32602703 PMC7368998

[9] C. Wu , T. J. Dougan , and D. R. Walt , “High‐Throughput, High‐Multiplex Digital Protein Detection With Attomolar Sensitivity,” ACS Nano 16 (2022): 1025–1035, 10.1021/acsnano.1c08675. 35029381 PMC9499451

[10] V. Yelleswarapu , J. R. Buser , M. Haber , J. Baron , E. Inapuri , and D. Issadore , “Mobile Platform for Rapid Sub–Picogram‐Per‐Milliliter, Multiplexed, Digital Droplet Detection of Proteins,” Proceedings of the National Academy of Sciences 116 (2019): 4489–4495, 10.1073/pnas.1814110116. PMC6410864 30765530

[11] S. J. Zhang , C. Wu , and D. R. Walt , “A Multiplexed Digital Platform Enables Detection of Attomolar Protein Levels With Minimal Cross‐Reactivity,” ACS Nano 18 (2024): 29891–29901, 10.1021/acsnano.4c10340. 39422558 PMC12914741

[12] J. Hu , J. Choy , J. S. Park , et al., “Fluorescence‐Coded, Magnetic Bead‐Enhanced Digital Proximity Extension Assay for Ultrasensitive, Multiplexed, and Accessible Protein Detection,” ACS Sensors 10 (2025): 8554–8565, 10.1021/acssensors.5c02315. 41092269 PMC13747061

[13] T. Gilboa , A. M. Maley , A. F. Ogata , C. Wu , and D. R. Walt , “Sequential Protein Capture in Multiplex Single Molecule Arrays: A Strategy for Eliminating Assay Cross‐Reactivity,” Advanced Healthcare Materials 10 (2021): 2001111, 10.1002/adhm.202001111. PMC8238389 32893488

[14] D. Juncker , S. Bergeron , V. Laforte , and H. Li , “Cross‐Reactivity in Antibody Microarrays and Multiplexed Sandwich Assays: Shedding Light on the Dark Side of Multiplexing,” Current Opinion in Chemical Biology 18 (2014): 29–37, 10.1016/j.cbpa.2013.11.012. 24534750

[15] J. P. Frampton , J. B. White , A. B. Simon , M. Tsuei , S. Paczesny , and S. Takayama , “Aqueous Two‐Phase System Patterning of Detection Antibody Solutions for Cross‐Reaction‐Free Multiplex ELISA,” Scientific Reports 4 (2014): 4878, 10.1038/srep04878. 24786974 PMC4007081

[16] M. Pla‐Roca , R. F. Leulmi , S. Tourekhanova , et al., “Antibody Colocalization Microarray: A Scalable Technology for Multiplex Protein Analysis in Complex Samples*,” Molecular & Cellular Proteomics 11 (2012): M111.011460, 10.1074/mcp.M111.011460. PMC3322566 22171321

[17] O. Poetz , T. Henzler , M. Hartmann , et al., “Sequential Multiplex Analyte Capturing for Phosphoprotein Profiling ,” Molecular & Cellular Proteomics 9 (2010): 2474–2481, 10.1074/mcp.M110.002709. 20682761 PMC2984240

[18] M. Dagher , G. Ongo , N. Robichaud , et al., “nELISA: A High‐Throughput, High‐Plex Platform Enables Quantitative Profiling of the Inflammatory Secretome,” Nature Methods 22 (2025): 2375–2385, 10.1038/s41592-025-02861-6. 41203862 PMC12615254

[19] W. Feng , J. C. Beer , Q. Hao , et al., “NULISA: A Proteomic Liquid Biopsy Platform With Attomolar Sensitivity and High Multiplexing,” Nature Communications 14 (2023): 7238, 10.1038/s41467-023-42834-x. PMC10636041 37945559

[20] M. Lundberg , S. B. Thorsen , E. Assarsson , et al., “Multiplexed Homogeneous Proximity Ligation Assays for High‐throughput Protein Biomarker Research in Serological Material*,” Molecular & Cellular Proteomics 10 (2011): M110.004978, 10.1074/mcp.M110.004978. PMC3069344 21242282

[21] M. Lundberg , A. Eriksson , B. Tran , E. Assarsson , and S. Fredriksson , “Homogeneous Antibody‐Based Proximity Extension Assays Provide Sensitive and Specific Detection of Low‐Abundant Proteins in Human Blood,” Nucleic Acids Research 39 (2011): e102–e102, 10.1093/nar/gkr424. 21646338 PMC3159481

[22] J. Candia , G. N. Daya , T. Tanaka , L. Ferrucci , and K. A. Walker , “Assessment of Variability in the Plasma 7k SomaScan Proteomics Assay,” Scientific Reports 12 (2022): 17147, 10.1038/s41598-022-22116-0. 36229504 PMC9561184

[23] F. Chowdhury , A. Williams , and P. Johnson , “Validation and Comparison of Two Multiplex Technologies, Luminex® and Mesoscale Discovery, for Human Cytokine Profiling,” Journal of Immunological Methods 340 (2009): 55–64, 10.1016/j.jim.2008.10.002. 18983846

[24] Y. Zhao , F. Chen , Q. Li , L. Wang , and C. Fan , “Isothermal Amplification of Nucleic Acids,” Chemical Reviews 115 (2015): 12491–12545, 10.1021/acs.chemrev.5b00428. 26551336

[25] J. Park , M. Feng , J. Yang , et al., “Agarose Microgel‐Based in Situ Cleavable Immuno‐Rolling Circle Amplification for Multiplexed Single‐Molecule Quantitation on Single Extracellular Vesicles,” ACS Nano 19 (2025): 17884–17899, 10.1021/acsnano.5c04207. 40320637 PMC12755089

[26] K. Sato , R. Ishii , N. Sasaki , K. Sato , and M. Nilsson , “Bead‐based Padlock Rolling Circle Amplification for Single DNA Molecule Counting,” Analytical Biochemistry 437 (2013): 43–45, 10.1016/j.ab.2013.02.016. 23467098

[27] E. Schopf , Y. Liu , J. C. Deng , S. Yang , G. Cheng , and Y. Chen , “Mycobacterium Tuberculosis Detection via Rolling Circle Amplification,” Analytical Methods 3 (2011): 267–273, 10.1039/C0AY00529K. 32938023

[28] W. Ouyang , S. Rutz , N. K. Crellin , P. A. Valdez , and S. G. Hymowitz , “Regulation and Functions of the IL‐10 Family of Cytokines in Inflammation and Disease,” Annual Review of Immunology 29 (2011): 71–109, 10.1146/annurev-immunol-031210-101312. 21166540

[29] M. W. L. Teng , E. P. Bowman , J. J. McElwee , et al., “IL‐12 and IL‐23 Cytokines: From Discovery to Targeted Therapies for Immune‐Mediated Inflammatory Diseases,” Nature Medicine 21 (2015): 719–729, 10.1038/nm.3895. 26121196

[30] S. A. Jones and B. J. Jenkins , “Recent Insights Into Targeting the IL‐6 Cytokine Family in Inflammatory Diseases and Cancer,” Nature Reviews Immunology 18 (2018): 773–789, 10.1038/s41577-018-0066-7. 30254251

[31] D. Wu , T. L. Dinh , B. P. Bausk , and D. R. Walt , “Long‐Term Measurements of Human Inflammatory Cytokines Reveal Complex Baseline Variations Between Individuals,” The American Journal of Pathology 187 (2017): 2620–2626, 10.1016/j.ajpath.2017.08.007. 28919109

[32] D. E. Leisman , L. Ronner , R. Pinotti , et al., “Cytokine Elevation in Severe and Critical COVID‐19: A Rapid Systematic Review, Meta‐Analysis, and Comparison With Other Inflammatory Syndromes,” Lancet Respiratory Medicine 8 (2020): 1233–1244, 10.1016/S2213-2600(20)30404-5. 33075298 PMC7567529

[33] C. Heeschen , S. Dimmeler , C. W. Hamm , et al., “Serum Level of the Antiinflammatory Cytokine Interleukin‐10 Is an Important Prognostic Determinant in Patients with Acute Coronary Syndromes,” Circulation 107 (2003): 2109–2114, 10.1161/01.CIR.0000065232.57371.25. 12668510

[34] T. Herold , V. Jurinovic , C. Arnreich , et al., “Elevated Levels of IL‐6 and CRP Predict the Need for Mechanical Ventilation in COVID‐19,” Journal of Allergy and Clinical Immunology 146 (2020): 128–136.e124, 10.1016/j.jaci.2020.05.008. 32425269 PMC7233239

[35] D. A. A. Vignali , “Multiplexed Particle‐Based Flow Cytometric Assays,” Journal of Immunological Methods 243 (2000): 243–255, 10.1016/S0022-1759(00)00238-6. 10986418

[36] S. Birtwell and H. Morgan , “Microparticle Encoding Technologies for High‐Throughput Multiplexed Suspension Assays,” Integrative Biology 1 (2009): 345–362, 10.1039/b905502a. 20023742 PMC7108550

[37] A. J. Rivnak , D. M. Rissin , C. W. Kan , et al., “A Fully‐Automated, Six‐Plex Single Molecule Immunoassay for Measuring Cytokines in Blood,” Journal of Immunological Methods 424 (2015): 20–27, 10.1016/j.jim.2015.04.017. 25960176 PMC10502417

[38] D. Briukhovetska , J. Dörr , S. Endres , P. Libby , C. A. Dinarello , and S. Kobold , “Interleukins in Cancer: From Biology to Therapy,” Nature Reviews Cancer 21 (2021): 481–499, 10.1038/s41568-021-00363-z. 34083781 PMC8173513

[39] G. Schett , B. McInnes Iain , and F. N. Markus , “Reframing Immune‐Mediated Inflammatory Diseases Through Signature Cytokine Hubs,” New England Journal of Medicine 385 (2021): 628–639, 10.1056/NEJMra1909094. 34379924

[40] B. Becher , S. Tugues , and M. Greter , “GM‐CSF: From Growth Factor to Central Mediator of Tissue Inflammation,” Immunity 45 (2016): 963–973, 10.1016/j.immuni.2016.10.026. 27851925

[41] F. Balkwill , “Tumour Necrosis Factor and Cancer,” Nature Reviews Cancer 9 (2009): 361–371, 10.1038/nrc2628. 19343034

[42] R. Drapkin , H. H. von Horsten , Y. Lin , et al., “Human Epididymis Protein 4 (HE4) Is a Secreted Glycoprotein That Is Overexpressed by Serous and Endometrioid Ovarian Carcinomas,” Cancer Research 65 (2005): 2162–2169, 10.1158/0008-5472.CAN-04-3924. 15781627

[43] V. Dochez , H. Caillon , E. Vaucel , J. Dimet , N. Winer , and G. Ducarme , “Biomarkers and Algorithms for Diagnosis of Ovarian Cancer: CA125, HE4, RMI and ROMA, A Review,” Journal of Ovarian Research 12 (2019): 28, 10.1186/s13048-019-0503-7. 30917847 PMC6436208

[44] K. Huhtinen , P. Suvitie , J. Hiissa , et al., “Serum HE4 Concentration Differentiates Malignant Ovarian Tumours From Ovarian Endometriotic Cysts,” British Journal of Cancer 100 (2009): 1315–1319, 10.1038/sj.bjc.6605011. 19337252 PMC2676558

[45] D. Trudel , B. Têtu , J. Grégoire , et al., “Human Epididymis Protein 4 (HE4) and Ovarian Cancer Prognosis,” Gynecologic Oncology 127 (2012): 511–515, 10.1016/j.ygyno.2012.09.003. 22967799

[46] L. Chang , D. M. Rissin , D. R. Fournier , et al., “Single Molecule Enzyme‐Linked Immunosorbent Assays: Theoretical Considerations,” Journal of Immunological Methods 378 (2012): 102–115, 10.1016/j.jim.2012.02.011. 22370429 PMC3327511

[47] D. M. Rissin , D. R. Fournier , T. Piech , et al., “Simultaneous Detection of Single Molecules and Singulated Ensembles of Molecules Enables Immunoassays With Broad Dynamic Range,” Analytical Chemistry 83 (2011): 2279–2285, 10.1021/ac103161b. 21344864 PMC3056883

[48] M. Dagher , M. Kleinman , A. Ng , and D. Juncker , “Ensemble Multicolour FRET Model Enables Barcoding at Extreme FRET Levels,” Nature Nanotechnology 13 (2018): 925–932, 10.1038/s41565-018-0205-0. 30061659

[49] Q. Wei , H. Qi , W. Luo , et al., “Fluorescent Imaging of Single Nanoparticles and Viruses on a Smart Phone,” ACS Nano 7 (2013): 9147–9155, 10.1021/nn4037706. 24016065 PMC3951925

[50] M. M. Hasan , M. W. Alam , K. A. Wahid , S. Miah , and K. E. Lukong , “A Low‐Cost Digital Microscope With Real‐Time Fluorescent Imaging Capability,” PLoS ONE 11 (2016): e0167863, 10.1371/journal.pone.0167863. 27977709 PMC5158004

[51] C. Coarsey , B. Coleman , M. A. Kabir , M. Sher , and W. Asghar , “Development of a Flow‐Free Magnetic Actuation Platform for an Automated Microfluidic ELISA,” RSC Advances 9 (2019): 8159–8168, 10.1039/C8RA07607C. 31777654 PMC6880949

[52] D. Liu , X. Li , J. Zhou , et al., “A Fully Integrated Distance Readout ELISA‐Chip for Point‐of‐Care Testing With Sample‐in‐Answer‐Out Capability,” Biosensors and Bioelectronics 96 (2017): 332–338, 10.1016/j.bios.2017.04.044. 28525851

[53] R. S. Sista , A. E. Eckhardt , V. Srinivasan , M. G. Pollack , S. Palanki , and V. K. Pamula , “Heterogeneous Immunoassays Using Magnetic Beads on a Digital Microfluidic Platform,” Lab on a Chip 8 (2008): 2188–2196, 10.1039/b807855f. 19023486 PMC2726047

